# Breakthrough seizures—Further analysis of the Standard versus New Antiepileptic Drugs (SANAD) study

**DOI:** 10.1371/journal.pone.0190035

**Published:** 2017-12-21

**Authors:** Laura J. Bonnett, Graham A. Powell, Catrin Tudur Smith, Anthony G. Marson

**Affiliations:** 1 Department of Biostatistics, University of Liverpool, Liverpool, Merseyside, United Kingdom; 2 Department of Molecular and Clinical Pharmacology, University of Liverpool, Liverpool, Merseyside, United Kingdom; Columbia University Medical Center, UNITED STATES

## Abstract

**Objectives:**

To develop prognostic models for risk of a breakthrough seizure, risk of seizure recurrence after a breakthrough seizure, and likelihood of achieving 12-month remission following a breakthrough seizure. A breakthrough seizure is one that occurs following at least 12 months remission whilst on treatment.

**Methods:**

We analysed data from the SANAD study. This long-term randomised trial compared treatments for participants with newly diagnosed epilepsy. Multivariable Cox models investigated how clinical factors affect the probability of each outcome. Best fitting multivariable models were produced with variable reduction by Akaike’s Information Criterion. Risks associated with combinations of risk factors were calculated from each multivariable model.

**Results:**

Significant factors in the multivariable model for risk of a breakthrough seizure following 12-month remission were number of tonic-clonic seizures by achievement of 12-month remission, time taken to achieve 12-month remission, and neurological insult. Significant factors in the model for risk of seizure recurrence following a breakthrough seizure were total number of drugs attempted to achieve 12-month remission, time to achieve 12-month remission prior to breakthrough seizure, and breakthrough seizure treatment decision. Significant factors in the model for likelihood of achieving 12-month remission after a breakthrough seizure were gender, age at breakthrough seizure, time to achieve 12-month remission prior to breakthrough, and breakthrough seizure treatment decision.

**Conclusions:**

This is the first analysis to consider risk of a breakthrough seizure and subsequent outcomes. The described models can be used to identify people most likely to have a breakthrough seizure, a seizure recurrence following a breakthrough seizure, and to achieve 12-month remission following a breakthrough seizure. The results suggest that focussing on achieving 12-month remission swiftly represents the best therapeutic aim to reduce the risk of a breakthrough seizure and subsequent negative outcomes. This will aid individual patient risk stratification and the design of future epilepsy trials.

## Introduction

Epilepsy is one of the most common serious neurological disorders worldwide, affecting approximately 50 million people. Estimates suggest that 60 to 70% of people with epilepsy will achieve a remission from seizures.[1] However, up to 37% of these people may proceed to have a breakthrough seizure.[2] A breakthrough seizure is defined as an epileptic seizure which occurs despite the use of antiepileptic drugs that have otherwise successfully prevented seizures in the patient.[3]

Breakthrough seizures might occur for a number of reasons–those inherent to the person’s epilepsy, or the natural history of the condition. Inherent factors include the dose of antiepileptic drug treatment being insufficient to reduce the seizure rate to zero, missed doses of medication, or provoking factors such as emotional stress, sleep deprivation, alcohol or other recreational drugs, and TV or video games.[4, 5] For some people, the natural history is to develop treatment refractoriness following a period of remission, presumably due to on-going epileptogenic processes.[6–8] Frequently, the cause of a breakthrough seizure may not be identified.

Some argue that breakthrough seizures are more dangerous than non-breakthrough seizures as they are unexpected by the patient, and therefore, the person may not take appropriate precautions.[9] Breakthrough seizures can have severe clinical consequences for the person–they may be admitted to hospital either as a result of the seizure, or because of injuries sustained during the seizure. Breakthrough seizures can take the form of status epilepticus which is associated with elevated morbidity, and potentially mortality.[10, 11]

Despite the fact that breakthrough seizures are commonly seen in clinical practice, very few publications have examined factors associated with a breakthrough seizure and outcomes following such a seizure. Two papers consider breakthrough seizures among people with epilepsy in developing countries,[12, 13] however similar papers for people in developed countries are lacking. It is clearly important that we are able to stratify for outcome following a breakthrough seizure to identify those likely to regain seizure control, and those with a worse prognosis who may need more intensive management. This analysis investigates the risk of a first breakthrough seizure following the first period of 12-month remission, the likelihood of a seizure recurrence following a breakthrough seizure, and the chance of achieving a period of 12-month remission following a breakthrough seizure. Included participants were those recruited to the UK-based multi-centre Standard versus New Antiepileptic Drug (SANAD) study.

## Methods

### Participants

Full details of the SANAD study have been published elsewhere.[14, 15] In brief, people were eligible for inclusion if they had a history of at least two clinically definite unprovoked epileptic seizures in the last year, and they were aged at least five years. Participants were recruited to Arm A if the recruiting clinician considered carbamazepine to be the standard treatment option. Between December 1^st^ 1999 and June 1^st^ 2001 participants were randomised in a ratio of 1:1:1:1 to carbamazepine, gabapentin, lamotrigine, or topiramate. From 1^st^ June 2001 to 31^st^ August 2004 an oxcarbazepine group was added to the trial.

People were recruited into Arm B if the recruiting clinician regarded valproate the standard treatment option. Participants were randomised in a 1:1:1 ratio to valproate, lamotrigine or topiramate between January 12^th^ 1999 and August 31^st^ 2004.

The two primary outcomes in SANAD were time to treatment failure from randomisation and time to the first period of 12-month remission from seizures following randomisation. In this paper the SANAD Arm A and SANAD Arm B datasets have been combined in order to undertake prognostic modelling, stratifying by study arm. In the original publications trial arms were analysed and reported separately, as the primary purpose was to compare the effectiveness of new antiepileptic drugs with the standard treatments. Here the purpose is different, the aim being to assess the risk of a breakthrough seizure, or outcome following a breakthrough seizure, irrespective of the specific drug that the patient was on at randomisation, or the subsequent choice of treatment.

Relevant participants for these analyses were those who had achieved their first period of 12-month remission whilst on treatment. No age or other restrictions were imposed.

SANAD received appropriate multicentre and local ethics and research committee approvals, and was managed according to the Medical Research Council’s Good Clinical Practice Guidelines. Patients gave informed written consent to inclusion and to long-term follow-up. SANAD is registered as an International Standard Randomised Controlled Trial, number ISRCTN38354748.

### Statistical analysis

The three outcomes of interest were (1) first breakthrough seizure following first period of 12-month remission, (2) seizure recurrence following a first breakthrough seizure, and (3) 12-month remission following a first breakthrough seizure. The risk estimates were all conditional on achieving a first period of 12-month remission.

Each outcome was analysed using a Cox proportional hazard model. For the risk of breakthrough seizure analysis, time zero was the time at which a 12-month period of seizure freedom was achieved. For example, if a participant had 23 months of seizure freedom immediately after randomisation followed by a seizure, their start time for the analysis of breakthrough seizure was 12 months, and their time to breakthrough seizure would be 11 months. For the other two outcomes, time zero was the date of the first breakthrough seizure following first period of 12-month remission.

Variables associated with a higher risk of seizure recurrence were determined univariably and after adjusting for multiple variables using log-rank tests and Cox proportional hazards modelling methods. Best fitting, parsimonious, multivariable models were produced with variable reduction by Akaike’s Information Criterion (AIC)–the model with the smallest AIC was identified as the parsimonious model.[16] Risk estimates for combinations of clinical risk factors were calculated from each multivariable model.[17] In particular, the baseline survivor function was estimated for each model, and then raised to a suitable power based on the combination of risk factors being considered.

Schöenfeld residual plots [18] and incorporation of time-dependent covariate effects were used to investigate the proportional hazards assumption. The predictive accuracy of the models was assessed using the c-statistic.[19] Analyses were performed using R version 3.2.3,[20] and significance was set at the 5% level. Computer code for all analyses are available in S1, S2 and S3 Files.

The list of potential prognostic factors for all three outcomes included: gender, febrile seizure history, first degree relative with epilepsy, neurological insult (learning difficulty or neurological deficit defined as localising neurological signs resulting in functional impairment), seizure type, epilepsy type, baseline electroencephalogram (EEG) result, baseline computerised tomography (CT) or magnetic resonance imaging (MRI) result, total number of treatments attempted to achieve first period of 12-month remission (one, or more than one), and time to achieve 12-month remission from randomisation. Additionally, total number of tonic-clonic seizures ever up until achievement of 12-month remission (classified according to the International League Against Epilepsy seizure classification[21]), and age at achievement of 12-month remission were considered in the risk of breakthrough seizure analysis. Total number of tonic-clonic seizures ever until breakthrough seizure, age at breakthrough seizure, and breakthrough seizure treatment decision (leave as it is, increase, or decrease) were additionally included in the analysis of seizure recurrence following a first breakthrough seizure, and 12-month remission following a first breakthrough seizure.

EEG was classified as normal, not done, non-specific abnormality, or epileptiform abnormality (focal or generalized spikes or spike and slow wave activity). Epilepsy type was classified as focal, generalised, or unclassified with the unclassified category being used when there was uncertainty between focal onset and generalised onset seizures.

Continuous variables (time to 12-month remission, total number of tonic-clonic seizures and age) were investigated using log and fractional polynomial transformations.[22–25] Results for the continuous variables are presented as post-hoc defined categorical variables with categories chosen according to knot positions for a spline model fit to the data.[26]

## Results

**Fig 1:**
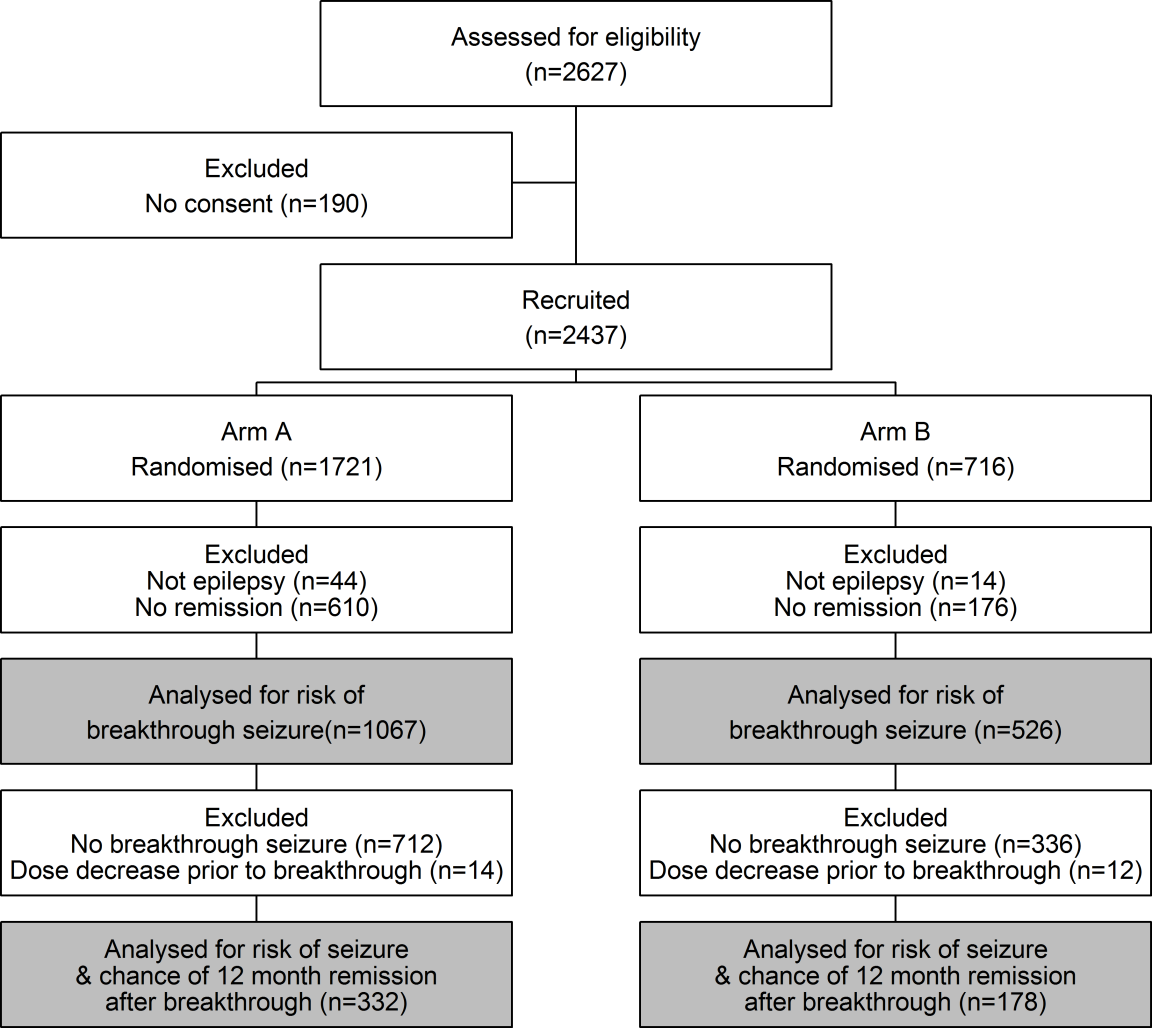
Trial profile.

Fig 1 illustrates the disposition of the 2627 participants recruited into both Arm A and Arm B of SANAD. It also identifies participants relevant to each of the outcomes in this analysis.

### Risk of a breakthrough seizure

**Fig 2:**
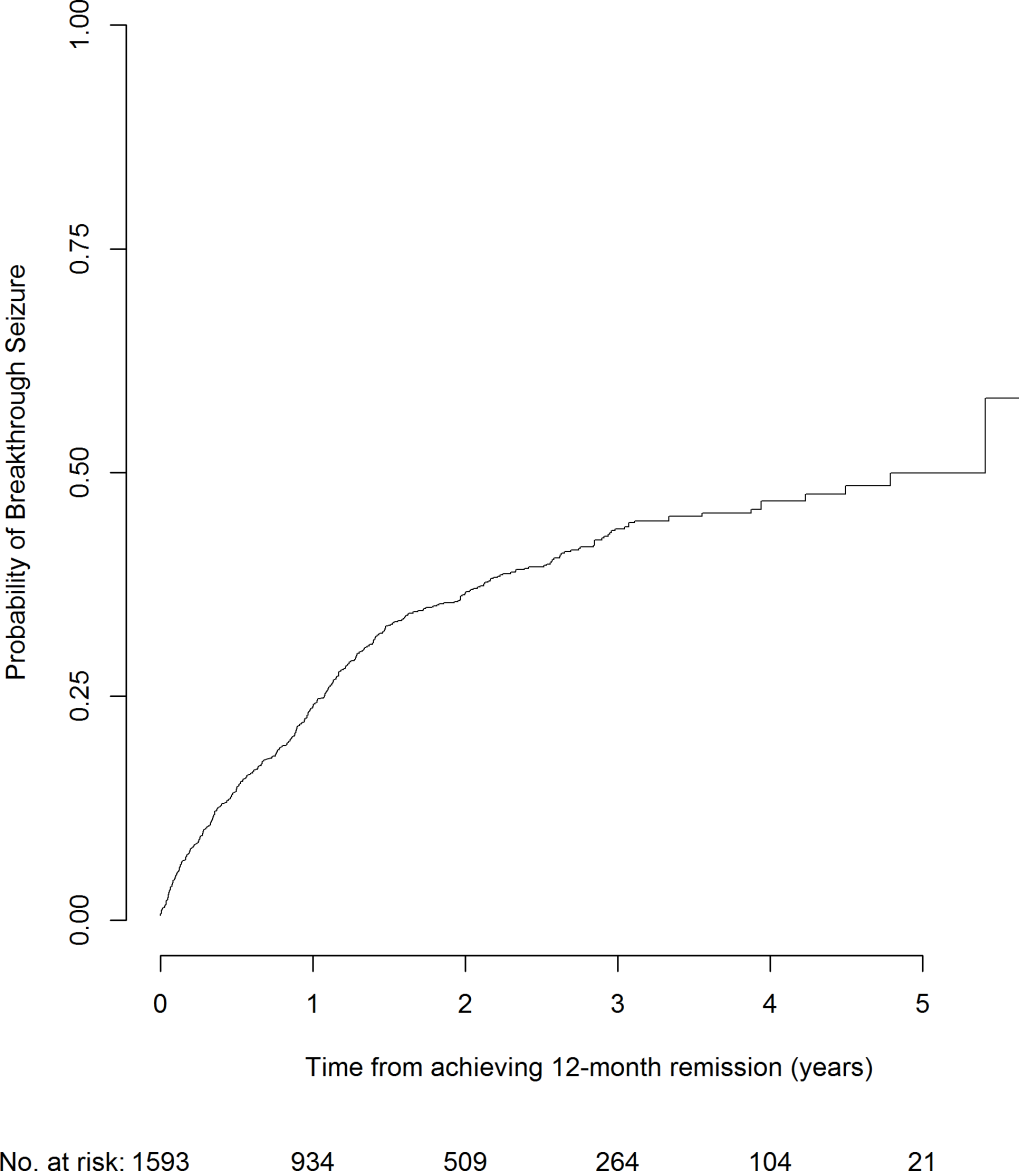
Risk of a first breakthrough seizure following a period of at least 12 months remission from seizures whilst on treatment.

Table 1 summarises the participant demographics for those achieving a first period of 12 month remission on treatment who were therefore at risk of a first breakthrough seizure. At 2 years following a remission, the overall risk of a breakthrough seizure is 37% (Fig 2). Of the 1593 participants included in this analysis, 536 had a first breakthrough seizure with median time to first breakthrough seizure 0.7 years from starting treatment (interquartile range (IQR) 0.2–1.2 years). Additionally, the median follow-up time from achievement of 12-month remission to date of last follow-up was 2.0 years (IQR 1.0–3.3 years).

**Table 1:** Participant demographics for those at risk of a first breakthrough seizure, n (%) unless otherwise stated.

Characteristic	Arm A (n = 1067)	Arm B (n = 526)	Total (n = 1593)
Male	609 (57)	314 (60)	923 (58)
Febrile Seizure History	61 (6)	44 (8)	105 (7)
Epilepsy in first degree relative	111 (10)	89 (17)	200 (13)
Neurological insult	104 (10)	52 (10)	156 (10)
Seizures			
Simple or Complex Partial with Secondary Generalised Seizures	597 (56)	15 (3)	622 (39)
Simple or Complex Partial only	328 (31)	25 (4)	343 (22)
Generalised tonic-clonic seizures only	15 (1)	154 (29)	169 (11)
Absence seizures	2 (0)	82 (16)	84 (5)
Myoclonic or absence seizures with tonic- clonic seizures	3 (0)	106 (20)	109 (7)
Tonic-clonic seizures, uncertain if focal or generalised	113 (11)	114 (22)	227 (14)
Other	9 (1)	30 (6)	39 (2)
Epilepsy type			
Partial	929 (87)	40 (8)	969 (61)
Generalised	21 (2)	357 (68)	378 (24)
Unclassified	117 (11)	129 (24)	246 (15)
EEG results			
Normal	472 (44)	129 (25)	601 (38)
Non-specific Abnormality	180 (17)	55 (10)	235 (15)
Epileptiform Abnormality	328 (31)	321 (61)	649 (40)
Not done^a^	87 (8)	21 (4)	108 (7)
CT/MRI scan results			
Normal	639 (60)	233 (44)	872 (55)
Abnormal	262 (25)	30 (6)	292 (18)
Not done^b^	166 (15)	263 (50)	429 (27)
Drugs attempted to achieve 12-month remission			
One	805 (75)	397 (75)	1202 (75)
Two or more	262 (25)	129 (25)	391 (25)
Number of tonic-clonic seizures ever from randomisation to achievement of 12-month remission, median (IQR)	2 (0, 4)	3 (1, 5)	2 (0, 5)
Age at achievement of 12-month remission (years), median (IQR)	38 (25, 55)	20 (14, 30)	31 (19, 49)
Time to achieve 12-month remission from randomisation (years), median (IQR)	1.1 (1.0, 1.8)	1.1 (1.0, 1.8)	1.1 (1.0, 1.8)

^a^Most of these patients had focal epilepsy

^b^Most of these patients were aged < 20 years

Results for multivariable modelling of risk of breakthrough seizure are presented in Table 2. Univariable results, including the log-rank test p-values, are available in S1 Table. The multivariable model included three covariates—neurological insult as recorded at randomisation, total number of tonic-clonic seizures recorded before achieving first period of 12-month remission, and time taken to achieve first period of 12-month remission following randomisation. Participants with neurological insult were more likely to have a first breakthrough seizure. Similarly, participants having one or more tonic-clonic seizures ever before achieving 12-month remission, and taking longer than 12 months to achieve their first period of 12-month remission were also at an increased risk of a first breakthrough seizure. The c-statistic for the model was 0.6, indicating that the model accurately discriminates participants 60% of the time, which is reasonable internal validation.[27, 28]

**Table 2:** Multivariable model hazard ratios for time to first breakthrough seizure after a period of 12-month remission whilst on treatment.

Variable	Comparison	Multivariable HR (95% CI)
Neurological insult as recorded at randomisation	Absent	1.00
	Present	*1*.*55 (1*.*21*, *1*.*98)*
Total number of tonic-clonic seizures recorded before achieving 12-month remission	0	1.00
	1	*1*.*03 (1*.*02*, *1*.*04)*
	2	*1*.*07 (1*.*04*, *1*.*10)*
	3–4	*1*.*10 (1*.*06*, *1*.*15)*
	5–6	*1*.*14 (1*.*07*, *1*.*20)*
	7–10	*1*.*17 (1*.*09*, *1*.*25)*
	11–20	*1*.*22 (1*.*12*, *1*.*33)*
	>20	*1*.*56 (1*.*28*, *1*.*90)*
Time taken to achieve 12-month remission following randomisation (years)	1	1.00
	1–1.5	*1*.*27 (1*.*16*, *1*.*39)*
	1.5–2	*1*.*56 (1*.*32*, *1*.*84)*
	2–3	*1*.*74 (1*.*42*, *2*.*14)*
	>3	*1*.*87 (1*.*49*, *2*.*36)*

HR>1 suggests breakthrough seizure more likely

Italic results are statistically significant

As can be seen in S1 Fig and S1 Table, at two years after achieving 12-month remission participants without neurological insult, with only one prior tonic-clonic seizure, and achieving remission immediately at 12 months had a 31% risk of a breakthrough seizure (95% confidence interval (CI): 28%-35%). Conversely, participants with neurological insult, with 20 prior tonic-clonic seizures and requiring three years to achieve 12-month remission had a 71% risk of a breakthrough seizure two years after achieving 12-month remission (95% CI: 61%-80%).

### Risk of seizure recurrence after a breakthrough seizure

**Fig 3:**
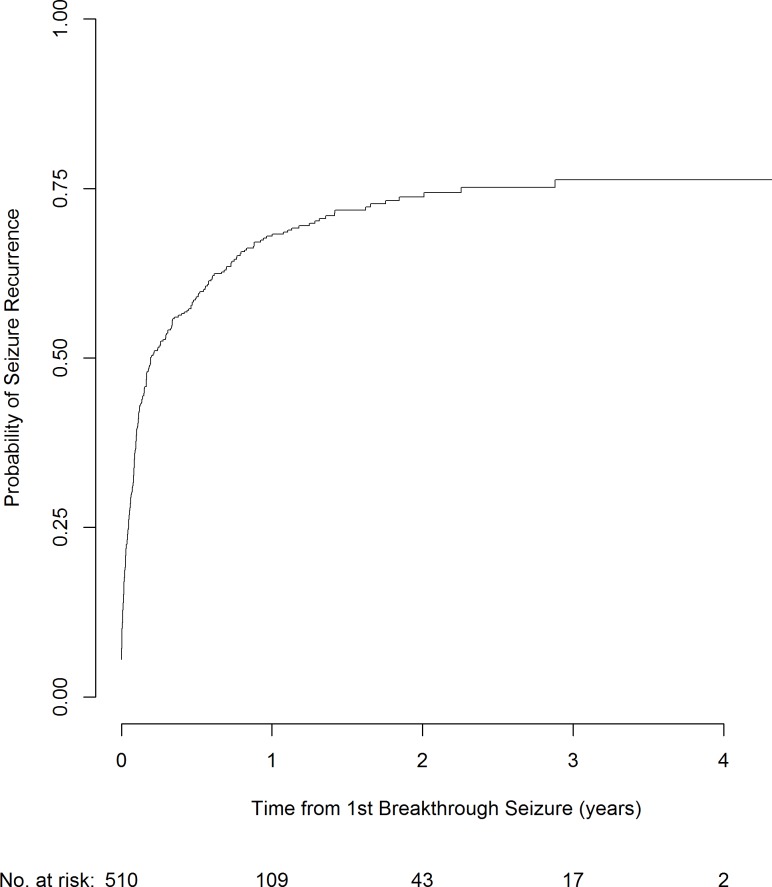
Risk of a seizure following a breakthrough seizure.

Table 3 summarises the participant demographics for those who have had a first breakthrough seizure following their first period of 12-month remission. These participants are consequently at risk of seizure recurrence, or have a chance of achieving a further period of 12-month remission. At 2 years following a first breakthrough seizure, the overall risk of a further seizure is 74% (Fig 3). Participants who were instructed to reduce their dose in the three months prior to their breakthrough seizure were removed from this analysis, irrespective of whether the reduction was with the intention to withdraw the drug or not.

**Table 3:** Participant demographics for those at risk of a seizure, or with a chance of achieving 12-month remission following a first breakthrough seizure, n (%) unless otherwise stated.

Characteristic	Arm A (n = 332)	Arm B (n = 178)	Total (n = 510)
Male	189 (57)	101 (57)	290 (57)
Febrile Seizure History	19 (6)	12 (7)	31 (6)
Epilepsy in first degree relative	29 (9)	38 (21)	67 (13)
Neurological insult	50 (15)	22 (12)	72 (14)
Seizures			
Simple/complex Partial + 2° generalised	198 (60)	6 (3)	204 (40)
Simple or complex partial only	88 (27)	6 (23)	94 (19)
Generalised tonic-clonic only	7 (1)	54 (30)	61 (12)
Absence	2 (1)	19 (11)	21 (4)
Myoclonic/absence + tonic- clonic seizures	2 (10)	44 (25)	46 (9)
Tonic-clonic (uncertain if focal or generalised)	30 (9)	37 (21)	67 (13)
Other	5 (2)	12 (7)	17 (3)
Epilepsy type			
Partial	287 (86)	12 (7)	299 (59)
Generalised	12 (4)	125 (70)	137 (27)
Unclassified	33 (10)	41 (23)	74 (14)
EEG results			
Normal	140 (42)	39 (22)	179 (35)
Non-specific Abnormality	53 (16)	19 (11)	72 (14)
Epileptiform Abnormality	105 (32)	116 (65)	221 (43)
Not done^a^	34 (10)	4 (2)	38 (8)
CT/MRI scan results			
Normal	186 (56)	75 (42)	261 (51)
Abnormal	86 (26)	12 (7)	98 (19)
Not done^b^	60 (18)	91 (51)	151 (30)
Drugs attempted to achieve 12-month remission			
One	252 (76)	136 (76)	388 (76)
Two or more	80 (24)	42 (24)	122 (24)
Number of tonic-clonic seizures ever until first breakthrough seizure, median (IQR)	2 (0, 6)	3 (2, 6)	3 (1, 6)
Age at first breakthrough seizure (years), median (IQR)	40.5 (24.1, 55.6)	20.7 (15.1, 26.3)	30.9 (19.0, 49.7)
Time to achieve 12-month remission from randomisation (years), median (IQR)	1.2 (1.0, 1.9)	1.2 (1.0, 1.8)	1.2 (1.0, 1.8)
Breakthrough seizure treatment decision			
No change to treatment plan	189 (59)	107 (63)	296 (60)
Increase dosage	124 (39)	62 (36)	186 (38)
Decrease dosage (or not specified)	9 (2)	2 (1)	11 (2)

^a^Most of these patients had focal epilepsy

^b^Most of these patients were aged < 20 years

Of the 510 participants included in this analysis, 322 people had a seizure recurrence with median time to seizure recurrence 30.9 days (IQR 6.5–93.3 days) from the first breakthrough seizure. Additionally, the median duration of follow-up time after first breakthrough seizure (following 12-month remission) was 1.6 years (IQR 0.8–2.6 years). The median number of seizures following the first breakthrough seizure was 1 (IQR 0–7). However, 45% participants have more than one seizure before re-entering 12-month remission.

Results for multivariable modelling of seizure recurrence after first breakthrough seizure are presented in Table 4. (Univariable results, including the log-rank test p-values, can be seen in S3 Table). The multivariable model included three variables–total number of drugs attempted to achieve initial period of 12-month remission, time to achieve first period of 12-month remission from randomisation, and treatment decision following first breakthrough seizure. Participants attempting two or more antiepileptic drugs to achieve first period of 12-month remission were more likely to have a seizure recurrence following a first breakthrough seizure than those requiring only one drug. Additionally, participants taking longer than one year to achieve an initial period of 12-month remission were more likely to have a recurrence following a first breakthrough seizure than those who only took a year. Participants who were told to increase their dose after their breakthrough seizure also had an increased chance of seizure recurrence compared to those who do not change their treatment plan. This may indicate that clinicians were able to identify participants with provoking factors or missed doses of medication which were the likely cause of the breakthrough seizure. The c-statistic for this model was 0.6, again showing reasonable internal validation.

**Table 4:** Effect estimates from multivariable models–risk of seizure recurrence following first breakthrough seizure (n = 510) and likelihood of achieving 12-month remission following a breakthrough seizure (n = 510).

		Multivariable HR (95% CI)	
Variable	Comparison	Seizure recurrence post breakthrough seizure	12-month remission post breakthrough seizure
Gender	Female	N/A	1.00
	Male		*1*.*34 (1*.*02*, *1*.*77)*
Drugs attempted to achieve 12-month remission	1	1.00	N/A
	2 or more	*1*.*47 (1*.*14*, *1*.*91)*	
Age at first breakthrough seizure (years)	≤ 20	N/A	1.00
	21–30		*0*.*92 (0*.*86*, *0*.*99)*
	31–45		*0*.*87 (0*.*78*, *0*.*98)*
	46–70		*0*.*83 (0*.*77*, *0*.*97)*
	> 70		*0*.*80 (0*.*66*, *0*.*96)*
Time to achieve 12-month remission (years)	1	1.00	1.00
	1–1.5	*1*.*03 (1*.*00*, *1*.*06)*	*0*.*90 (0*.*84*, *0*.*95)*
	1.5–2	*1*.*08 (1*.*01*, *1*.*15)*	*0*.*72 (0*.*60*, *0*.*87)*
	2–3	*1*.*13 (1*.*01*, *1*.*26)*	*0*.*52 (0*.*36*, *0*.*76)*
	>3	*1*.*22 (1*.*02*, *1*.*45)*	*0*.*22 (0*.*09*, *0*.*52)*
Breakthrough seizure decision	No change to treatment plan	1.00	1.00
	Increase dosage	*2*.*05 (1*.*63*, *2*.*57)*	*0*.*63 (0*.*47*, *0*.*84)*
	Decrease dosage (or not specified)	1.02 (0.58, 1.80)	0.61 (0.32, 1.16)

HR>1 implies greater chance of seizure recurrence or greater chance of 12-month remission following a breakthrough seizure as relevant

Italic results are statistically significant

Rates of seizure recurrence predicted by the model at 0.5 and 1 year after a first breakthrough seizure can be seen in S2 Fig and S4 Table. The data show that treatment decision following the breakthrough seizure has the biggest effect on risk of recurrence. The effect of number of drugs attempted to achieve initial period of 12-month remission is noticeable, whilst the time to achieve inital period of 12-month remission has a smaller effect.

### Chance of achieving 12-month remission after a breakthrough seizure

**Fig 4:**
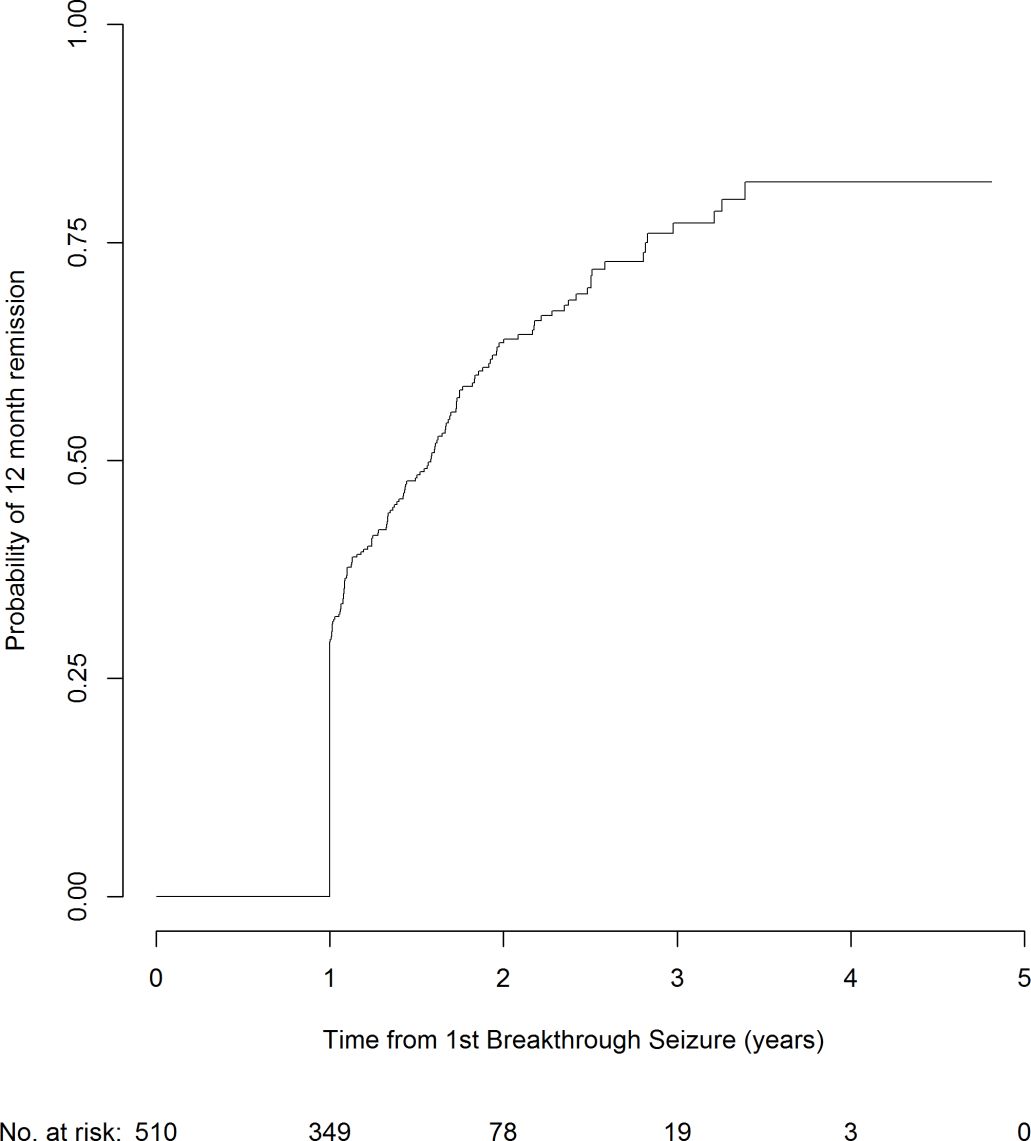
Chance of achieving 12 month remission following a breakthrough seizure.

Of the 510 participants included in this analysis, 223 people went on to achieve 12-month remission following a first breakthrough seizure, with median time to seizure recurrence 1.0 years (IQR 1.0–1.6 years). At 2 years following a breakthrough seizure, the overall chance of re-entering a period of 12-month remission is 64% (Fig 4).

Results for multivariable modelling of chance of 12-month remission following a first breakthrough seizure are presented in Table 4 (univariable results in S3 Table). The multivariable model included gender, age at first breakthrough seizure, time to achieve first period of 12-month remission, and treatment decision following first breakthrough seizure. According to the model, men are 34% more likely than women to achieve 12-month remission after a first breakthrough seizure. Participants achieving their initial period of 12-month remission immediately after randomisation were more likely to achieve a 12-month remission after a first breakthrough seizure than those taking longer than one year to achieve remission. Participants who did not change their dose after their breakthrough seizure were more likely to achieve a 12-month remission after a first breakthrough seizure than those who increased their dose. Additionally, participants who were less than or equal to 20 years old were more likely to have a 12-month remission after a first breakthrough seizure than those aged over 20. The c-statistic for this model was again 0.6.

The range of likelihoods of achieiving 12-month remission predicted by the model at 1 and 2 years after a first breakthrough seizure are shown in S3 Fig and S5 Table. The data show that time to achieve initial period of 12-month remission has the biggest effect on chance of achieving 12-month remission following a first breakthrough seizure. The effect of treatment decision following the breakthrough seizure is also very clear. The effect of age at acheivement of initial period of 12-month remission is noticable, whilst gender has a smaller effect.

## Discussion

We have shown that several clinical factors influence the risk of a first breakthrough seizure following an initial period of 12-month remission whilst on treatment, and outcomes following such a seizure. Of the participants recruited into SANAD, 34% went on to have a first breakthrough seizure. According to the multivariable model for this outcome, participants with neurological insult, or with any number of tonic-clonic seizures, or taking over a year to achieve initial period of 12-month remission were at increased risk of a first breakthrough seizure.

Of those participants who had a first breakthrough seizure, 63% went on to have seizure recurrence. The factor with the largest effect was antiepileptic drug treatment decision following the first breakthrough seizure. Those with no change were at much lower risk of a further seizure than those with a treatment increase. This might at first appear counterintuitive, but it may indicate that clinicians are able to identify seizures occurring as a result of participant non-adherence. Alternative reasons may include the presence of other lifestyle factors associated with increased seizure risk. The appropriate management for this perceived non-adherence is to recommend adherence with no dose change, or avoidance of other seizure provoking factors. However the clinician may not be aware of the presence of this non-adherence or provoking factors and may resultantly increase the antiepileptic drug dosage–a dose increase is usually indicated for those with a breakthrough seizure despite adhering to treatment and with no other seizure provoking factors. Other risk factors for this outcome were number of drugs required to achieve initial period of 12-month remission, and time to achieve first period of 12-month remission—participants requiring polytherapy to achieve first period of 12-month remission and taking longer than one year to achieve it were more likely to have a recurrence.

Of participants who had a first breakthrough seizure, 44% went on to achieve another period of 12-month remission. Male participants, participants aged under 20 years, and participants achieving their first period of 12-month remission immediately at one year after randomisation were significantly more likely to achieve 12-month remission following a first breakthrough seizure. This gender effect was also observed for the primary outcomes in the SANAD trial, whereby men were more likely to achieve an initial period of 12-month remission.[29] This effect remains unexplained, and might have a biological explanation, or may be because men might be less likely to report seizures in order to minimise impact on their employment or driving license. Participants with no recommended antiepileptic drug treatment change after a first breakthrough seizure were also more likely to achieve 12-month remission than those who increased their dose, indicating (as discussed above) that clinicians might be able to identify those with seizures due to provoking factors requiring no dose change.

Our model for risk of a first breakthrough seizure is the first known analysis of risk factors for a first breakthrough seizure in developed countries. However, the results are broadly in line with those published considering risk factors for treatment failure following randomisation to the SANAD study.[30, 31] The Arm A multivariable model focussed on participants with focal epilepsy and included variables for gender, treatment history, age, total number of seizures prior to randomisation, EEG result, seizure type, focal epilepsy site of onset, and randomised treatment. Of these, only number of tonic-clonic seizures was in common with the model presented in this paper. The Arm B multivariable model focussed on participants with generalised and unclassified epilepsy and included variables for treatment history, EEG result, seizure type, and randomised treatment.

Previous work also considered risk of second treatment failure after a first and the likelihood of achieving 12-month remission following a treatment failure.[32] The multivariable model for second treatment failure included covariates for total number of tonic-clonic seizures before first treatment failure, reasons for treatment failure, and CT/MRI scan result. The multivariable model for likelihood of achieving 12-month remission following a treatment failure included covariates for gender, age, time on randomised treatment at first treatment failure, neurological insult, total number of tonic-clonic seizure before first treatment failure, reason for treatment failure, seizure type, and CT/MRI scan result.[32]

### Limitations

Pragmatic clinical trials usually recruit a heterogeneous group of participants. Although some have criticised this approach [33, 34] the strength of this method has been highlighted here as it allows an investigation of sources of heterogeneity of outcome. Other limitations of SANAD have been discussed elsewhere.[29]

EEG was not included in the final model which was selected based on statistical model selection methods. However, EEG was undertaken at randomisation rather than at the time of the breakthrough seizure–measuring EEG at time of breakthrough would have significant resource implications for health services. Additionally, adherence was not measured and therefore could not be included in the list of covariates for possible inclusion in any model. However, no affordable methods exist at present to measure adherence in long-term pragmatic publically funded trials.

Due to the definition of each end point—particularly the post breakthrough seizure endpoints—the sample size is relatively small. Additionally, due to the extended follow-up period required to observe participants having events of interest, the duration of follow-up after a first breakthrough is quite limited. These two factors potentially reduce the power of the analyses and could mean that some significant results are not identified.

This manuscript has presented a number of models that can further inform participant counselling and potentially treatment decision making. However, these models require validation in other similar datasets. The predictive power of each model also needs to be explored. SANAD II is currently underway. In the meantime there are no other datasets that are similar to SANAD. The closest match is a set of individual participant data collected by the authors.[35] This data is however missing important covariates. Internal validation of the models presented here suggests reasonable model fit however.

### Conclusions

This is the first analysis to consider the risk of a breakthrough seizure and outcomes following a breakthrough seizure, in participants from a developed country. The SANAD Study is currently the largest and longest study of participants with newly diagnosed epilepsy and therefore provides the best evidence for this work.

Participants taking a long time to achieve first period of 12-month remission, and having a large number of seizures, are the most likely to have a first breakthrough seizure. However once a first breakthrough seizure has occurred only time to achieve initial period of 12-month remission continues to be important. Instead, number of drugs required to achieve initial period of remission, gender and age are found to be associated with the outcomes. Therefore, a focus on achieving 12-month remission swiftly represents the best therapeutic aim to reduce the risk of a first breakthrough seizure and subsequent negative outcomes.

## Supporting information

S1 Table Univariable model hazard ratios for time to breakthrough seizure after a period of 12 month remission whilst on treatment. (DOCX)

S2 Table Numerical results for combinations of risk factors for chance of breakthrough seizure following 12 months remission at 1, 2 and 3 years after achieving remission. (DOCX)

S3 Table Effect estimates from univariable models–risk of seizure recurrence following first breakthrough seizure and likelihood of achieving 12 month remission following a breakthrough seizure. (DOCX)

S4 Table Numerical results for combinations of risk factors for risk of seizure following a breakthrough seizure at 0.5 and 1 year after a breakthrough seizure. (DOCX)

S5 Table Numerical results for combinations of risk factors for chance of 12 month remission following a breakthrough seizure at 1 and 2 years after a breakthrough seizure. (DOCX)

S1 Fig Combinations of risk factors for chance of breakthrough seizure following 12 months remission at 1, 2 and 3 years after achieving remission. (TIF)

S2 Fig Forest-style plot for risk of seizure following a breakthrough seizure at 0.5 and 1 year after a breakthrough seizure. (TIF)

S3 Fig Forest-style plot for chance of 12-month remission following a breakthrough seizure at 1 and 2 years after a breakthrough seizure. (TIF)

S1 File R code for estimating risk of breakthrough seizure. (TXT)

S2 File R code for estimating risk of second seizure following a first breakthrough seizure. (TXT)

S3 File R code for estimating chance of 12-month remission following a first breakthrough seizure. (TXT)
