# Supplementary material for: Breakthrough seizures—Further analysis of the Standard versus New Antiepileptic Drugs (SANAD) study
Source: PLoS One. 2017 Dec 21;12(12):e0190035. doi: 10.1371/journal.pone.0190035 (PMC5739445; doi:10.1371/journal.pone.0190035)
Supplement: S1 Table — (DOCX) [file pone.0190035.s001.docx]

**S1 Table**

| **Variable** | **Comparison** | **Univariable**  **p-value** | **Univariable HR (95% CI)** |
| --- | --- | --- | --- |
| Gender | Female | 0.71 | 1.00 |
|  | Male |  | 0.97 (0.82, 1.15) |
| Febrile Seizure History | Absent | 0.76 | 1.00 |
|  | Present |  | 0.95 (0.67, 1.34) |
| Epilepsy in 1^st^ degree relative | Absent | 0.65 | 1.00 |
|  | Present |  | 1.06 (0.82, 1.37) |
| Neurological insult | Absent | *<0.001* | 1.00 |
|  | Present |  | *1.67 (1.31, 2.14)* |
| Seizures | Simple/complex partial + 2° gen |  | 1.00 |
|  | Simple/complex partial only | 0.18 | 0.85 (0.67, 1.08) |
|  | Generalised TC only | 0.68 | 1.09 (0.73, 1.62) |
|  | Absence | 0.21 | 0.72 (0.42, 1.21) |
|  | Myoclonic/absence + TC | 0.17 | 1.34 (0.88, 2.06) |
|  | TC (uncertain if focal or generalised) | 0.51 | 0.90 (0.67, 1.22) |
|  | Other | 0.16 | 1.48 (0.86, 2.56) |
| Epilepsy type | Partial |  | 1.00 |
|  | Generalised | 0.36 | 1.18 (0.83, 1.67) |
|  | Unclassified | 0.89 | 0.98 (0.74, 1.30) |
| EEG results | Normal |  | 1.00 |
|  | Non-specific Abnormality | 0.40 | 1.12 (0.86, 1.47) |
|  | Epileptiform Abnormality | 0.06 | 1.21 (0.99, 1.48) |
|  | Not done | *0.05* | *1.41 (1.01, 1.97)* |
| CT/MRI scan results | Normal |  | 1.00 |
|  | Abnormal | 0.45 | 1.09 (0.87, 1.38) |
|  | Not done | *0.04* | *1.25 (1.01, 1.53)* |
| Total number of drugs attempted to achieve 12 month remission | 1 | *0.04* | 1.00 |
|  | 2 or more |  | *1.23 (1.01, 1.50)* |
| Number of tonic-clonic seizures ever until achievement of 12 month remission | 0 | *<0.001* | 1.00 |
|  | 1 |  | *1.03 (1.02, 1.05)* |
|  | 2 |  | *1.08 (1.05, 1.11)* |
|  | 3-4 |  | *1.11 (1.07, 1.16)* |
|  | 5-6 |  | *1.15 (1.09, 1.21)* |
|  | 7-10 |  | *1.18 (1.10, 1.26)* |
|  | 11-20 |  | *1.23 (1.13, 1.33)* |
|  | >20 |  | *1.57 (1.31, 1.89)* |
| Age at achievement of 12 month remission | ≤ 20 | 0.14 | 1.00 |
|  | 21-30 |  | 0.97 (0.93, 1.01) |
|  | 31-45 |  | 0.95 (0.89, 1.02) |
|  | 46-70 |  | 0.93 (0.84, 1.02) |
|  | > 70 |  | 0.91 (0.81, 1.03) |
| Time to achieve 12 month remission (years) | 1 | *<0.001* | 1.00 |
|  | 1-1.5 |  | *1.30 (1.19, 1.42)* |
|  | 1.5-2 |  | *1.64 (1.39, 1.94)* |
|  | 2-3 |  | *1.85 (1.50, 2.28)* |
|  | >3 |  | *2.01 (1.59, 2.54)* |

Hazard Ratio (HR) >1 suggests breakthrough seizure more likely

Italic text is statistically significant
