# Supplementary material for: Breakthrough seizures—Further analysis of the Standard versus New Antiepileptic Drugs (SANAD) study
Source: PLoS One. 2017 Dec 21;12(12):e0190035. doi: 10.1371/journal.pone.0190035 (PMC5739445; doi:10.1371/journal.pone.0190035)
Supplement: S2 Table — (DOCX) [file pone.0190035.s002.docx]

**S2 Table**

| **Neurological insult** | **Number of tonic-clonic seizures**  **prior to achieving 12 month remission** | **Time taken to achieve**  **12 month remission (years)** | **1 Year: HR (95% CI)** | **2 Year: HR (95% CI)** | **3 Year: HR (95% CI)** |
| --- | --- | --- | --- | --- | --- |
| Absent | 1 | 1 | 20 (17, 22) | 31 (28, 35) | 38 (34, 42) |
| Absent | 1 | 2 | 30 (27, 34) | 46 (41, 51) | 55 (49, 60) |
| Absent | 1 | 3 | 33 (28, 37) | 49 (43, 55) | 58 (52, 64) |
| Absent | 5 | 1 | 21 (18, 24) | 33 (30, 37) | 41 (36, 45) |
| Absent | 5 | 2 | 32 (28, 36) | 49 (44, 54) | 58 (52, 63) |
| Absent | 5 | 3 | 35 (30, 40) | 52 (46, 58) | 61 (54, 68) |
| Absent | 20 | 1 | 23 (20, 27) | 36 (31, 41) | 44 (38, 49) |
| Absent | 20 | 2 | 35 (30, 39) | 52 (46, 58) | 61 (55, 67) |
| Absent | 20 | 3 | 37 (32, 43) | 55 (48, 62) | 64 (57, 71) |
| Present | 1 | 1 | 29 (23, 36) | 44 (36, 53) | 53 (44, 62) |
| Present | 1 | 2 | 43 (35, 51) | 61 (52, 70) | 71 (61, 79) |
| Present | 1 | 3 | 46 (37, 54) | 65 (55, 74) | 74 (64, 82) |
| Present | 5 | 1 | 31 (24, 38) | 47 (38, 56) | 55 (46, 65) |
| Present | 5 | 2 | 45 (37, 54) | 64 (55, 73) | 73 (64, 82) |
| Present | 5 | 3 | 48 (40, 57) | 68 (58, 77) | 77 (67, 85) |
| Present | 20 | 1 | 33 (26, 41) | 50 (41, 59) | 59 (49, 69) |
| Present | 20 | 2 | 48 (40, 57) | 67 (58, 77) | 76 (67, 85) |
| Present | 20 | 3 | 51 (42, 61) | 71 (61, 80) | 79 (70, 88) |

HR – Hazard Ratio
