# Supplementary material for: Breakthrough seizures—Further analysis of the Standard versus New Antiepileptic Drugs (SANAD) study
Source: PLoS One. 2017 Dec 21;12(12):e0190035. doi: 10.1371/journal.pone.0190035 (PMC5739445; doi:10.1371/journal.pone.0190035)
Supplement: S3 Table — (DOCX) [file pone.0190035.s003.docx]

**S3 Table**

|  |  | **Seizure recurrence post breakthrough seizure** | | **12 month remission post breakthrough seizure** | |
| --- | --- | --- | --- | --- | --- |
| **Variable** | **Comparison** | **p-value** | **Univariable HR (95% CI)** | **p-value** | **Univariable HR (95% CI)** |
| Gender | Female | 0.45 | 1.00 | 0.08 | 1.00 |
|  | Male |  | 1.09 (0.87, 1.36) |  | 1.28 (0.97, 1.67) |
| Febrile Seizure History | Absent | 0.28 | 1.00 | 0.49 | 1.00 |
|  | Present |  | 0.76 (0.44, 1.29) |  | 1.21 (0.70, 2.09) |
| Epilepsy in 1^st^ degree relative | Absent | 0.76 | 1.00 | 0.74 | 1.00 |
|  | Present |  | 1.06 (0.75, 1.50) |  | 0.94 (0.63, 1.38) |
| Neurological insult | Absent | 0.69 | 1.00 | 0.78 | 1.00 |
|  | Present |  | 0.94 (0.69, 1.28) |  | 1.05 (0.74, 1.49) |
| Seizure type | Simple/complex partial + 2° gen. |  | 1.00 |  | 1.00 |
|  | Simple/complex partial only | 0.12 | 1.26 (0.94, 1.69) | 0.31 | 0.82 (0.56, 1.21) |
|  | Generalised TC only | 0.29 | 0.77 (0.48, 1.25) | 0.09 | 1.60 (0.93, 2.76) |
|  | Absence | 0.23 | 0.65 (0.33, 1.31) | 0.81 | 1.10 (0.52, 2.33) |
|  | Myoclonic/absence + TC | 0.20 | 0.68 (0.39, 1.22) | 0.98 | 0.99 (0.52, 1.90) |
|  | TC (uncertain if focal or gen.) | 0.05 | 0.66 (0.43, 1.01) | 0.29 | 1.28 (0.81, 2.02) |
|  | Other | 0.27 | 0.67 (0.33, 1.36) | *0.01* | *2.50 (1.21, 5.20)* |
| Epilepsy type | Partial |  | 1.00 |  | 1.00 |
|  | Generalised | 0.06 | 0.66 (0.43, 1.02) | 0.18 | 1.38 (0.86, 2.21) |
|  | Unclassified | *0.03* | *0.64 (0.44, 0.95)* | 0.09 | 1.45 (0.95, 2.21) |
| EEG results | Normal |  | 1.00 |  | 1.00 |
|  | Non-specific Abnormality | 0.49 | 0.88 (0.62, 1.25) | 0.34 | 1.24 (0.80, 1.90) |
|  | Epileptiform Abnormality | 0.59 | 0.93 (0.73, 1.20) | 0.18 | 1.24 (0.90, 1.71) |
|  | Not done/Missing | 0.05 | 0.60 (0.36, 1.01) | 0.25 | 1.36 (0.80, 2.31) |
| CT/MRI scan results | Normal |  | 1.00 |  | 1.00 |
|  | Abnormal | 0.41 | 0.88 (0.66, 1.19) | 0.80 | 0.95 (0.66, 1.38) |
|  | Not done/Missing | 0.62 | 0.93 (0.72, 1.22) | 0.14 | 1.26 (0.93, 1.73) |
| Total number of drugs attempted to achieve 12 month remission | 1 | *0.002* | 1.00 | 0.66 | 1.00 |
|  | 2 or more |  | *1.47 (1.15, 1.88)* |  | 0.93 (0.68, 1.28) |
| Number of tonic-clonic seizures reported ever by first breakthrough seizure | 0 | 0.51 | 1.00 | 0.92 | 1.00 |
|  | 1 |  | 1.00 (1.00, 1.00) |  | 1.00 (0.98, 1.02) |
|  | 2 |  | 1.00 (1.00, 1.01) |  | 1.00 (0.96, 1.05) |
|  | 3-4 |  | 1.00 (0.99, 1.01) |  | 1.00 (0.94, 1.07) |
|  | 5-6 |  | 1.00 (0.99, 1.02) |  | 1.00 (0.92, 1.10) |
|  | 7-10 |  | 1.00 (0.99, 1.02) |  | 1.01 (0.90, 1.12) |
|  | 11-20 |  | 1.01 (0.98, 1.04) |  | 1.01 (0.88, 1.15) |
|  | >20 |  | 1.36 (0.54, 3.44) |  | 1.01 (0.76, 1.35) |
| Age at first breakthrough seizure | ≤ 20 | 0.42 | 1.00 | *0.02* | 1.00 |
|  | 21-30 |  | 1.03 (0.96, 1.11) |  | *0.92 (0.86, 0.99)* |
|  | 31-45 |  | 1.06 (0.92, 1.23) |  | *0.88 (0.78, 0.98)* |
|  | 46-70 |  | 1.12 (0.85, 1.46) |  | *0.83 (0.71, 0.97)* |
|  | > 70 |  | 1.17 (0.79, 1.73) |  | *0.80 (0.66, 0.97)* |
| Time to achieve 12 month remission (years) | 1 | *0.003* | 1.00 | *<0.001* | 1.00 |
|  | 1-1.5 |  | *1.04 (1.01, 1.06)* |  | *0.90 (0.84, 0.96)* |
|  | 1.5-2 |  | *1.10 (1.03, 1.17)* |  | *0.73 (0.60, 0.88)* |
|  | 2-3 |  | *1.17 (1.05, 1.29)* |  | *0.53 (0.36, 0.77)* |
|  | >3 |  | *1.29 (1.09, 1.52)* |  | *0.22 (0.09, 0.54)* |
| Breakthrough seizure decision | No change to treatment plan |  | 1.00 |  | 1.00 |
|  | Increase dosage | *<0.001* | *1.99 (1.58, 2.49)* | *0.01* | *0.68 (0.51, 0.91)* |
|  | Decrease dosage (or not specified) | 0.75 | 1.10 (0.62, 1.93) | 0.13 | 0.60 (0.32, 1.15) |

Italic text is statistically significant; HR – Hazard Ratio
