# Supplementary material for: Breakthrough seizures—Further analysis of the Standard versus New Antiepileptic Drugs (SANAD) study
Source: PLoS One. 2017 Dec 21;12(12):e0190035. doi: 10.1371/journal.pone.0190035 (PMC5739445; doi:10.1371/journal.pone.0190035)
Supplement: S4 Table — (DOCX) [file pone.0190035.s004.docx]

**S4 Table**

| **Number of drugs attempted to achieve remission** | **Post breakthrough seizure treatment decision** | **Time taken to achieve**  **12 month remission (years)** | **0.5 Year: HR (95% CI)** | **1 Year: HR (95% CI)** |
| --- | --- | --- | --- | --- |
| 1 | Increase | 1 | 17 (3, 64) | 22 (4, 74) |
| 1 | Increase | 2 | 19 (4, 65) | 24 (5, 74) |
| 1 | Increase | 3 | 20 (4, 65) | 25 (6, 75) |
| 1 | No Change | 1 | 9 (2, 39) | 11 (2, 48) |
| 1 | No Change | 2 | 9 (2, 40) | 12 (3, 48) |
| 1 | No Change | 3 | 10 (2, 40) | 13 (3, 48) |
| 2 or more | Increase | 1 | 25 (5, 81) | 31 (6, 89) |
| 2 or more | Increase | 2 | 27 (5, 82) | 33 (7, 89) |
| 2 or more | Increase | 3 | 28 (6, 82) | 35 (8, 89) |
| 2 or more | No Change | 1 | 13 (2, 55) | 16 (3, 65) |
| 2 or more | No Change | 2 | 14 (3, 56) | 18 (4, 65) |
| 2 or more | No Change | 3 | 15 (3, 56) | 19 (4, 66) |

HR – Hazard Ratio
