# Supplementary material for: Breakthrough seizures—Further analysis of the Standard versus New Antiepileptic Drugs (SANAD) study
Source: PLoS One. 2017 Dec 21;12(12):e0190035. doi: 10.1371/journal.pone.0190035 (PMC5739445; doi:10.1371/journal.pone.0190035)
Supplement: S5 Table — (DOCX) [file pone.0190035.s005.docx]

**S5 Table**

| **Gender** | **Post breakthrough seizure treatment decision** | **Age (years)** | **Time taken to achieve**  **Initial 12 month remission (years)** | **1 Year: HR (95% CI)** | **2 Year: HR (95% CI)** |
| --- | --- | --- | --- | --- | --- |
| Female | Increase | 10 | 1 | 38 (23, 57) | 81 (60, 95) |
| Female | Increase | 10 | 2 | 26 (15, 43) | 65 (44, 86) |
| Female | Increase | 10 | 3 | 18 (9, 34) | 49 (28, 75) |
| Female | Increase | 30 | 1 | 33 (21, 48) | 75 (57, 90) |
| Female | Increase | 30 | 2 | 23 (14, 36) | 59 (41, 78) |
| Female | Increase | 30 | 3 | 15 (8, 28) | 44 (26, 67) |
| Female | Increase | 60 | 1 | 30 (20, 43) | 71 (55, 86) |
| Female | Increase | 60 | 2 | 21 (13, 32) | 55 (39, 73) |
| Female | Increase | 60 | 3 | 14 (8, 24) | 40 (24, 62) |
| Female | No Change | 10 | 1 | 53 (35, 73) | 93 (78, 99) |
| Female | No Change | 10 | 2 | 38 (24, 58) | 81 (61, 95) |
| Female | No Change | 10 | 3 | 27 (14, 47) | 66 (42, 89) |
| Female | No Change | 30 | 1 | 47 (33, 64) | 89 (75, 97) |
| Female | No Change | 30 | 2 | 34 (22, 49) | 76 (58, 90) |
| Female | No Change | 30 | 3 | 23 (13, 39) | 60 (39, 82) |
| Female | No Change | 60 | 1 | 44 (31, 58) | 86 (73, 95) |
| Female | No Change | 60 | 2 | 31 (21, 44) | 72 (56, 86) |
| Female | No Change | 60 | 3 | 21 (12, 35) | 56 (37, 77) |
| Male | Increase | 10 | 1 | 47 (30, 67) | 89 (71, 98) |
| Male | Increase | 10 | 2 | 33 (20, 52) | 76 (54, 92) |
| Male | Increase | 10 | 3 | 23 (12, 42) | 60 (36, 85) |
| Male | Increase | 30 | 1 | 41 (28, 58) | 84 (68, 95) |
| Male | Increase | 30 | 2 | 29 (19, 44) | 70 (51, 86) |
| Male | Increase | 30 | 3 | 20 (11, 35) | 54 (33, 77) |
| Male | Increase | 60 | 1 | 38 (27, 52) | 81 (67, 92) |
| Male | Increase | 60 | 2 | 27 (18, 39) | 66 (49, 82) |
| Male | Increase | 60 | 3 | 18 (10, 31) | 50 (32, 72) |
| Male | No Change | 10 | 1 | 64 (44, 83) | 97 (87, 100) |
| Male | No Change | 10 | 2 | 48 (31, 69) | 90 (72, 98) |
| Male | No Change | 10 | 3 | 34 (19, 57) | 77 (52, 95) |
| Male | No Change | 30 | 1 | 57 (42, 74) | 95 (85, 99) |
| Male | No Change | 30 | 2 | 42 (29, 59) | 85 (69, 96) |
| Male | No Change | 30 | 3 | 30 (17, 49) | 71 (48, 90) |
| Male | No Change | 60 | 1 | 54 (40, 68) | 93 (83, 98) |
| Male | No Change | 60 | 2 | 39 (27, 53) | 82 (67, 93) |
| Male | No Change | 60 | 3 | 27 (16, 44) | 67 (46, 86) |

HR – Hazard Ratio
