# Supplementary figures and images for: Breakthrough seizures—Further analysis of the Standard versus New Antiepileptic Drugs (SANAD) study
Source: PLoS One. 2017 Dec 21;12(12):e0190035. doi: 10.1371/journal.pone.0190035 (PMC5739445; doi:10.1371/journal.pone.0190035)

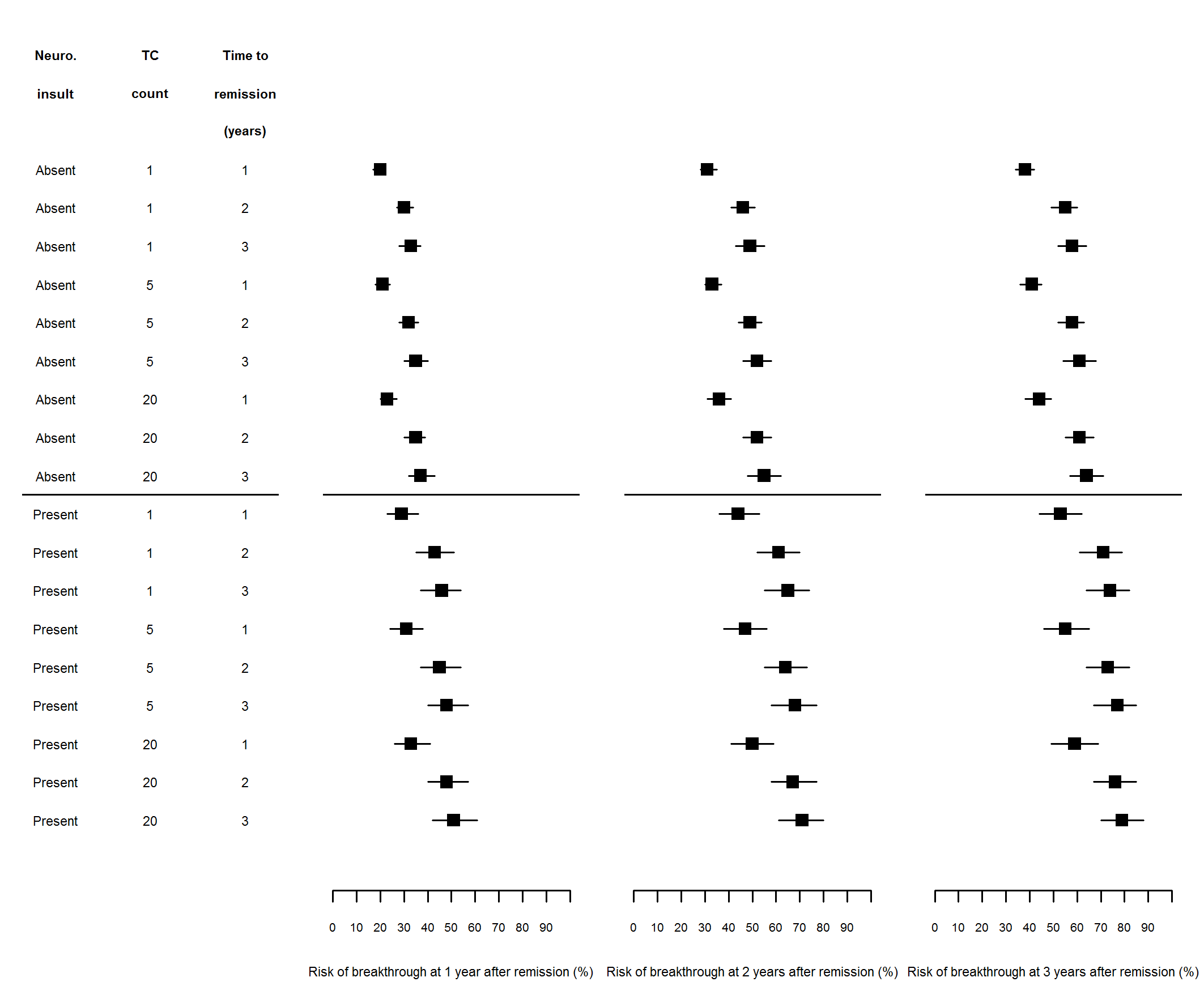

Supplement: S1 Fig — (TIF) [file pone.0190035.s006.tif]

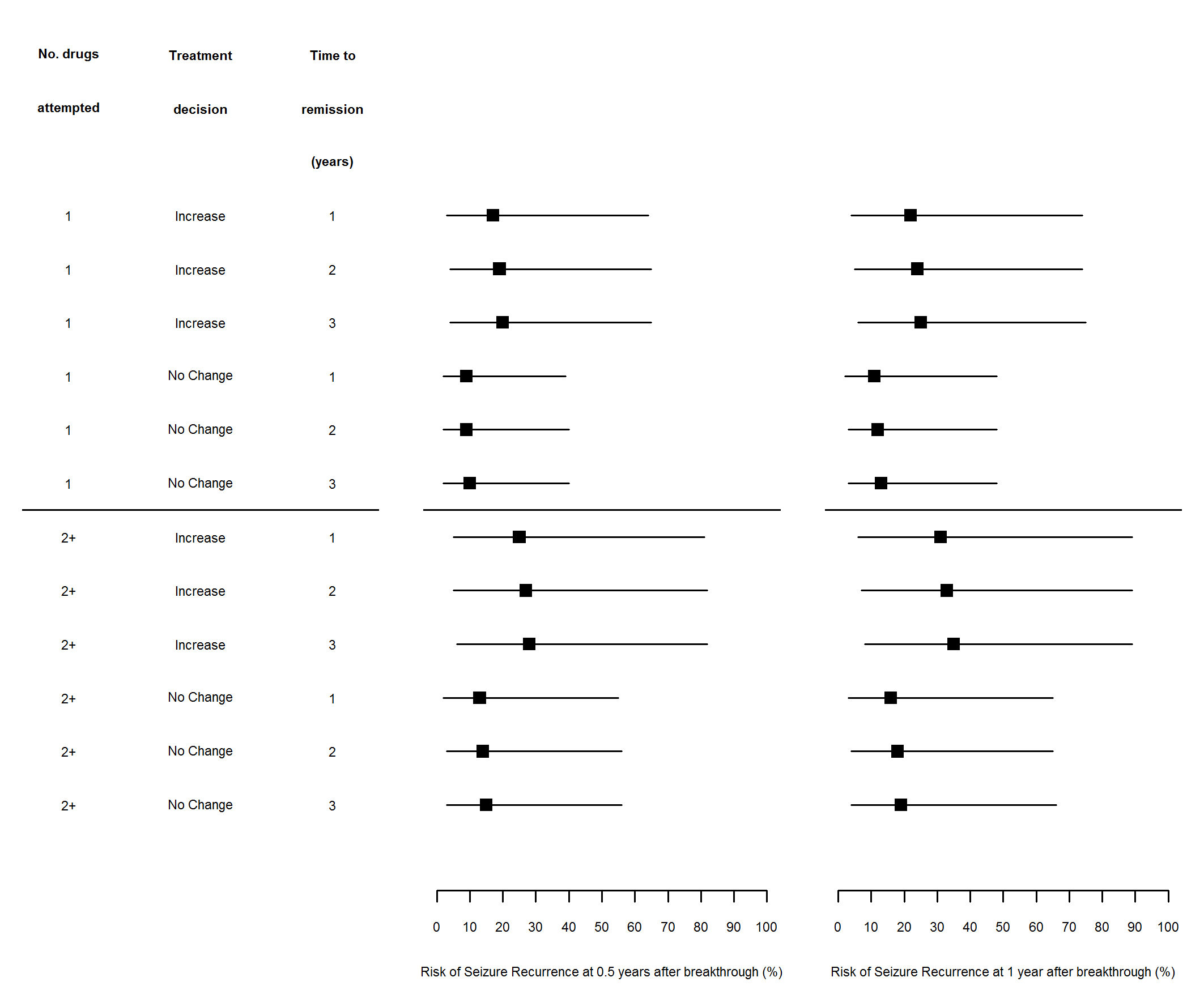

Supplement: S2 Fig — (TIF) [file pone.0190035.s007.tif]

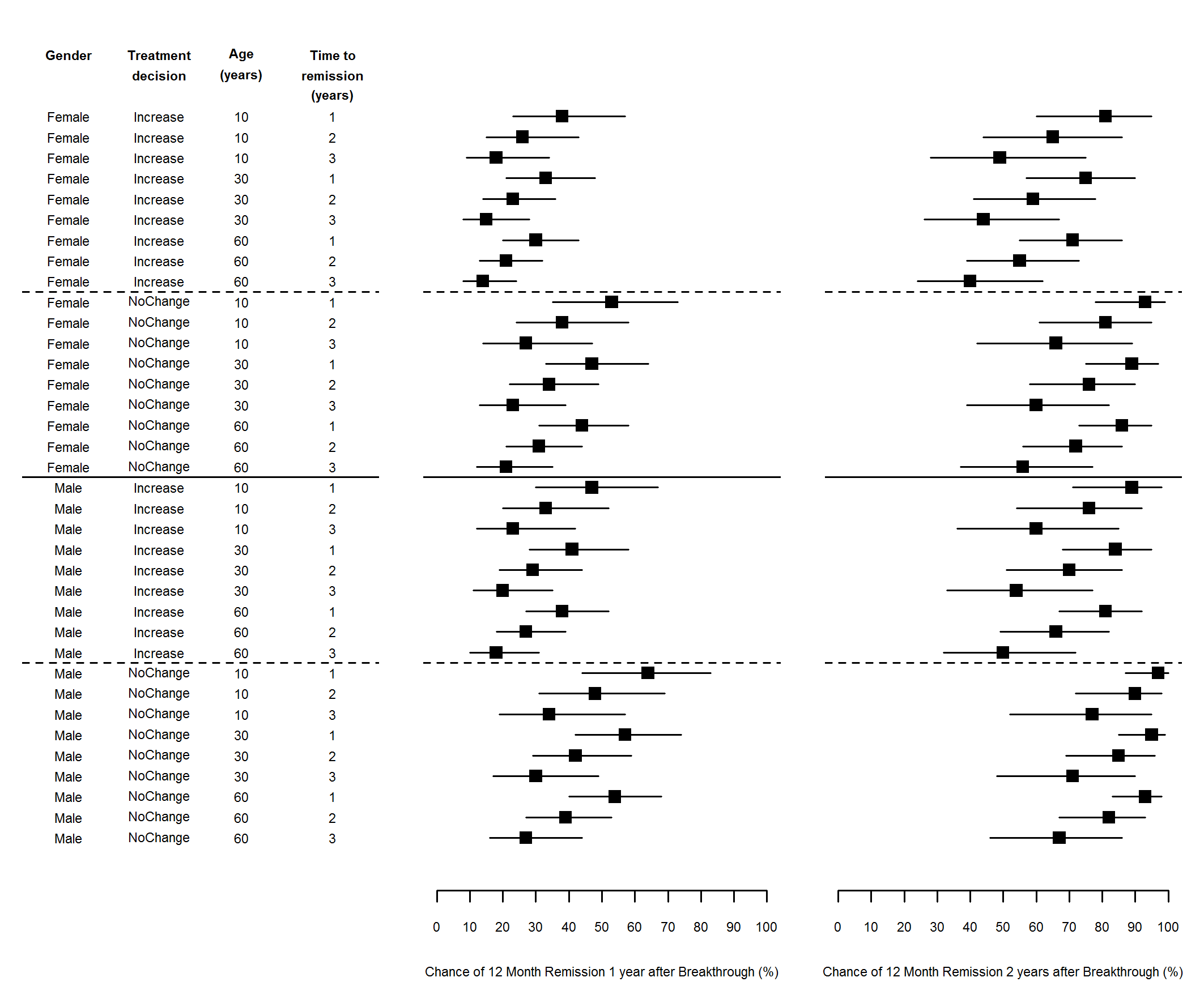

Supplement: S3 Fig — (TIF) [file pone.0190035.s008.tif]
